# Diagnostic Accuracy of the INSHI Consensus Case Definition for the Diagnosis of Paradoxical Tuberculosis-IRIS

**DOI:** 10.1097/QAI.0000000000002606

**Published:** 2020-12-17

**Authors:** Cari Stek, Jozefien Buyze, Joris Menten, Charlotte Schutz, Friedrich Thienemann, Lisette Blumenthal, Gary Maartens, Tom Boyles, Robert J. Wilkinson, Graeme Meintjes, Lutgarde Lynen

**Affiliations:** a Department of Clinical Sciences, Institute of Tropical Medicine, Antwerp, Belgium;; b Wellcome Centre for Infectious Diseases Research in Africa, Institute of Infectious Disease and Molecular Medicine, University of Cape Town, Cape Town, South Africa;; c Department of Medicine, University of Cape Town, Cape Town, South Africa;; d Department of Medicine, Imperial College London, London, United Kingdom; and; e The Francis Crick Institute, London, United Kingdom.

**Keywords:** tuberculosis, HIV, immune reconstitution inflammatory syndrome, TB-IRIS, latent class analysis

## Abstract

**Background:**

The diagnosis of paradoxical tuberculosis–associated immune reconstitution inflammatory syndrome (TB-IRIS) relies on characteristic clinical features synthesized as the International Network for the Study of HIV-associated IRIS (INSHI) case definition. There is no confirmatory laboratory test.

**Setting:**

Site B HIV-TB clinic in Khayelitsha, Cape Town, South Africa.

**Methods:**

Using data of participants with HIV-associated tuberculosis starting antiretroviral treatment from a prospective trial evaluating prednisone for TB-IRIS prevention, we applied latent class analysis to model a gold standard for TB-IRIS. The model-predicted probability of TB-IRIS for each participant was used to assess the performance of the INSHI case definition and compare its diagnostic accuracy with several adapted case definitions.

**Results:**

Data for this analysis were complete for 217 participants; 41% developed TB-IRIS. Our latent class model included the following parameters: respiratory symptoms; night sweats; INSHI major criteria 1, 2, and 4; maximum C-reactive protein >90 mg/L; maximum heart rate >120/min; maximum temperature >37.7°C; and preantiretroviral therapy CD4 count <50 cells/µL. The model estimated a TB-IRIS incidence of 43% and had optimal goodness of fit (χ^2^ = 337, *P* = 1.0). The INSHI case definition displayed a sensitivity of 0.77 and a specificity of 0.86. Replacing all the minor INSHI criteria with objectives measures (C-reactive protein elevation, fever, and/or tachycardia) resulted in a definition with better diagnostic accuracy, with a sensitivity of 0.89 and a specificity of 0.88.

**Conclusion:**

The INSHI case definition identifies TB-IRIS with reasonable accuracy. Amending the case definition by replacing INSHI minor criteria with objective variables improved sensitivity without loss of specificity.

## Abstract 2

Supplemental Digital Content is Available in the Text.

## INTRODUCTION

Paradoxical tuberculosis–associated immune reconstitution inflammatory syndrome (TB-IRIS) is an immunopathological reaction resulting in new or recurrent TB signs and symptoms, shortly after starting antiretroviral therapy (ART). It complicates treatment of HIV-associated tuberculosis (TB) in approximately 18% of cases, with rates more than 50% in high risk groups, causing significant morbidity.^1,2^ The diagnosis of TB-IRIS is largely based on its characteristic clinical presentation; there is no confirmatory laboratory test. The International Network for the Study of HIV-associated IRIS (INSHI) consensus case definition is used to diagnose TB-IRIS.^3^ It requires a diagnosis of TB with an initial positive response to treatment, characteristic clinical features (such as new or enlarging lymphadenopathy, constitutional, respiratory or abdominal symptoms, or new or worsening radiological features), and exclusion of alternative explanations for clinical deterioration—see Supplemental Digital Content, http://links.lww.com/QAI/B582. It is designed for use by both clinicians and researchers in a variety of settings and has been validated in several studies.^4–6^ However, in the absence of a gold standard, validation was always performed against diagnostic assignment based on expert opinion. Having a gold standard for TB-IRIS would allow for a more objective validation of the INSHI case definition and assessment of the ability of new variables to improve its diagnostic accuracy.

Latent class analysis (LCA) is a modeling technique that can identify unobserved groups, or classes, in a population. It has been used and shown utility in several diseases characterized by the lack of a gold standard, such as latent TB or sepsis.^7–11^ LCA divides the population in 2 groups: those who have the disease and those who do not have the disease. In the absence of a diagnostic standard for the disease, the disease itself cannot be measured directly. Instead, many clinical signs and symptoms exist, that are each in themselves imperfect predictors of the disease. These signs and symptoms cluster in different patterns in different individuals. The observed frequencies of these patterns allow for the construction of models estimating the prevalence of the disease and the sensitivity and specificity of each sign or symptom, or combinations thereof.

We used LCA to generate a surrogate gold standard for TB-IRIS. Next, we used the LCA-predicted probability of TB-IRIS for each participant to assess the performance of the INSHI case definition and compare its diagnostic accuracy with several adapted case definitions. We assessed the ability of additional variables to improve the diagnostic accuracy of the case definition. Finally, we assessed the ability of C-reactive protein (CRP) as a rule-out test for TB-IRIS.

## METHODS

### Main Trial and Setting

We performed a retrospective analysis of data collected in the PredART trial.^12^ This randomized, double-blind, placebo-controlled trial showed that a 28-day course of prophylactic prednisone in adult patients identified as being at a high risk (time between initiation of antituberculosis treatment and ART < 30 days; CD4 count **≤**100 cells/µL) for paradoxical TB-IRIS reduced the incidence of paradoxical TB-IRIS, without an excess of adverse events. Between August 2013 and February 2016, the trial enrolled 240 participants from Khayelitsha, Cape Town, South Africa. Their median age was 36, 60% were men, and their median CD4 count was 49 cells/µL. The primary endpoint of the trial was the development of TB-IRIS, which was adjudicated by a committee of 3 clinical experts not active at the clinical site who were given access to all clinical, laboratory data, and digital chest radiographs (CXR) and reports of other radiographic studies. They also accessed TB-IRIS narrative summaries that were written by the trial doctor for any clinical deterioration after initiation of ART. After completion of the trial, committee members independently assessed all available information from each participant who experienced clinical deterioration after initiation of ART. The INSHI consensus case definition was used to diagnose TB-IRIS (INSHI TB-IRIS). In addition, committee members were given the option to indicate when they considered that a participant developed TB-IRIS, although not fulfilling the INSHI case definition (paradoxical TB-IRIS not fulfilling the INSHI criteria); these participants were included in the analyses and defined as not having TB-IRIS according to INSHI criteria. Cases where there was disagreement, were resolved by consensus between the members.

### Selection of Variables

As variables for our latent class model, we included the following: baseline participant characteristics (sex, age, and details about current and previous TB episodes); INSHI major criteria; all individual symptoms of the INSHI minor criteria; any additionally documented signs and symptoms provided they occurred in more than 10 (4%) participants and could feasibly be related to TB-IRIS; baseline (immediately before starting ART) and follow-up laboratory variables (hemoglobin, leucocytes, creatinine clearance, alanine transferase, alkaline phosphatase, CRP, CD4 count, and HIV viral load); signs and symptoms that were identified as possibly related to TB-IRIS during the TB-IRIS adjudication process; and baseline urine lipoarabinomannan (LAM), determined retrospectively on stored urine using Alere Determine lateral flow assay.

Data for variables included were restricted to the first 4 weeks after ART initiation (95% of the TB-IRIS cases in our cohort occurred within 4 weeks, with a median time between ART initiation and TB-IRIS of 8 (interquartile range 5–13) days).

In LCA as we applied it, variables need to be binary; a sign or symptom was either present or absent in the first 4 weeks. Therefore, continuous variables were transformed into binary ones by plotting them in a receiver operating characteristics (ROC) curve and—after excluding variables with ROC area under the curve <0.55—selecting a cut-off that obtained the maximum of the sum of the computed sensitivities and specificities. For these variables to be positive, the highest [or, for hemoglobin (Hb) the lowest] value of that variable measured anytime during the first 4 weeks needed to be higher (lower for Hb) than the cut-off.

Given the relatively small number of participants in the study, only a limited number of variables could be included in the LCA model to avoid overfitting the model to the data.

To select which variables to include in the latent class model, we first calculated the unadjusted odds ratio (OR) for each individual variable and the consensus decision of the adjudication committee for INSHI TB-IRIS, using Stata version 14.2. These are listed in Table 1. We combined the abdominal symptoms associated with TB-IRIS into one new variable, that comprised “abdominal pain with either hepatomegaly, splenomegaly, or abdominal lymph nodes, or abdominal tenderness on clinical examination without other explanation.” We combined cough, dyspnea, and chest pain into the variable “respiratory symptoms.” Both new variables did not affect the selection of any other variable for inclusion in the latent class model.

**TABLE 1.** Variables Initially Included in the Selection for the Latent Class Model, Their Unadjusted Odds Ratio for Paradoxical INHSI IRIS, and Their Occurrence in Participants With and Without Paradoxical TB-IRIS According to the INSHI Consensus Case Definition

	Unadjusted Odds Ratio for INSHI IRIS (n = 238)	INSHI-IRIS (n = 89)	No INSHI-IRIS (n = 128)
Baseline variables			
Sex (M)	0.8	56 (63%)	76 (59%)
Age (<40 y)	2.1	67 (75%)	76 (59%)
Previous tuberculosis	0.9	8 (9%)	14 (11%)
Microbiological evidence of tuberculosis	1.8	71 (80%)	89 (70%)
Time from start of tuberculosis treatment to start of ART (<14d)	0.9	85 (96%)	89 (70%)
Baseline CD4 count (<50 cells/µL)	3.5	70 (79%)	62 (48%)
Baseline HIV viral load (<150,000 cp/mL)	1.0	12 (14%)	38 (30%)
Baseline urine LAM (≥1)	4.0	62 (73%)	45 (38%)
INSHI-related variables			
Major criteria			
Enlarged lymph nodes	—	30 (34%)	0 (0%)
New/worsening radiological features	—	40 (45%)	0 (0%)
CNS TB-IRIS*	—	0 (0%)	0 (0%)
New/worsening serositis	—	3 (3%)	0 (0%)
Minor criteria			
Weight loss†	4.0	45 (51%)	27 (21%)
Fever‡	8.7	33 (37%)	8 (6%)
Night sweats	8.4	50 (56%)	14 (11%)
Anorexia	3.2	47 (53%)	32 (25%)
Weakness	3.8	30 (34%)	13 (10%)
Cough	3.9	32 (36%)	16 (13%)
Chest pain	6.4	24 (27%)	8 (6%)
Dyspnea	8.3	22 (25%)	4 (3%)
Abdominal pain with hepatomegaly, splenomegaly, or enlarged abdominal lymph nodes	4.3	8 (9%)	3 (2%)
Included other adverse events§			
Headache	3.8	22 (25%)	11 (9%)
Dizziness	0.5	6 (7%)	10 (13%)
Arthralgia	1.1	6 (7%)	8 (6%)
Back pain	0.6	4 (4%)	8 (6%)
Flank pain	1.3	5 (6%)	5 (4%)
Epigastric pain	1.5	5 (6%)	5 (4%)
Abdominal pain	4.5	20 (22%)	8 (6%)
Nausea	2.8	27 (30%)	17 (13%)
Vomiting	2.5	34 (38%)	25 (20%)
Diarrhea‖	4.3	28 (31%)	9 (7%)
*Herpes simplex* infection	2.2	17 (19%)	13 (10%)
Pruritis	1.1	6 (7%)	7 (5%)
Paresthesia	0.9	7 (8%)	10 (8%)
Papular pruritic eruption	0.4	3 (3%)	11 (9%)
Variables raised during TB-IRIS adjudication			
Tachycardia (HR > 120/min)	4.9	62 (70%)	39 (30%)
Return of any (non-INSHI) initial tuberculosis symptom	2.5	21 (24%)	12 (9%)
Abdominal pain and tenderness without any other explanation	14.9	9 (10%)	1 (1%)
Raised CRP	5.7	55 (62%)	27 (21%)
Laboratory variables¶			
Hemoglobin (<10 g/dL)	2.0	52 (58%)	51 (39%)
Leukocytes (>10 × 10^9^ cells/L)	1.0	33 (37%)	11 (9%)
CRP (>90 mg/L)	5.7	55 (62%)	27 (21%)

Unadjusted ORs were computed including all participants of the PredART trial who had taken at least one dose of ART. Comparison between participants with and without paradoxical TB-IRIS was performed in participants included in the latent class model.

* None of the participants developed neurological TB-IRIS.

† Weight loss is defined as > 2.5% in 2 weeks or > 5% in 4 weeks.

‡ Fever is defined as temperature > 37.7°C, following the DAIDS table for grading the severity of adverse events that was current during the collection of data.

§ The following adverse events were excluded from analysis because of an unlikely association with TB-IRIS: upper respiratory tract infection (including coryza), blocked nose, ear symptoms, tooth ache, heartburn, hemorrhoids, swollen feet, a patient history of feeling hot or cold, scabies, urinary tract infection, and low potassium.

║ This includes both diarrhea and “more frequent than normal loose stools.”

¶ The following laboratory variables were excluded from analysis because the area under the curve in the receiver-operating characteristic curve was < 0.55: creatinine clearance, alanine transferase, alkaline phosphatase, increase in CD4 count at week 12, and decrease in HIV viral load at week 12.

We assessed different unadjusted ORs as cut-off for variable inclusion in the multivariable model: OR >2.0 or <0.5 (2-model), OR >3.0 or <0.3 (3-model), and OR >5.0 or <0.2 (5-model). We chose the 3-model because it was the most parsimonious.

We did not include urine LAM in the initial latent class model because of a higher number of participants with missing data (n = 35). Rather, we used a model containing urine LAM as an alternative model to compare findings with our final model.

Next, we combined variables selected based on their unadjusted OR in a backward multiple logistic regression model. Those variables with a *P*-value < 0.1 were retained and entered in the latent class model, together with the INSHI major criteria. The LCA model was limited to a maximum of 10 variables to avoid too many variable combinations with no or very few observations. This limits the number of observations to 9 per variable, in line with the recommendation to limit the number of variables in a logistic regression model to 1 per 10 cases.^13^

### Latent Class Model

We performed LCA using LEM software [J. Vermunt, Tilburg, The Netherlands, 1993; https://jeroenvermunt.nl]. We assumed the presence of 2 latent classes, corresponding to TB-IRIS and no TB-IRIS. We initially fitted models applying the conditional independence assumption. Goodness of fit tests—using the χ^2^ test—confirmed there was no requirement to include conditional dependencies between variables in the LCA model. We excluded 21 participants with missing data on any of the selected variables because LEM does not allow missing values. Baseline variables for these participants were comparable with those of the included participants (data not shown).

### Diagnostic Accuracy of Various Case Definitions and CRP

After running the latent class model, we used the LCA-predicted probability of TB-IRIS for each participant to first calculate the positive and negative predictive values for each sign or symptom initially considered for LCA, followed by their sensitivity and specificity, again using Stata version 14.2. We combined the individual signs and symptoms to match the INSHI consensus case definition or form several adapted case definitions. Doing so, we could assess the performance of the INSHI case definition and compare its diagnostic accuracy with the adapted case definitions. We also assessed the diagnostic ability of CRP to rule-out TB-IRIS, using a range of lower cut-off values, aiming for a high sensitivity.

## RESULTS

**FIGURE 1.**
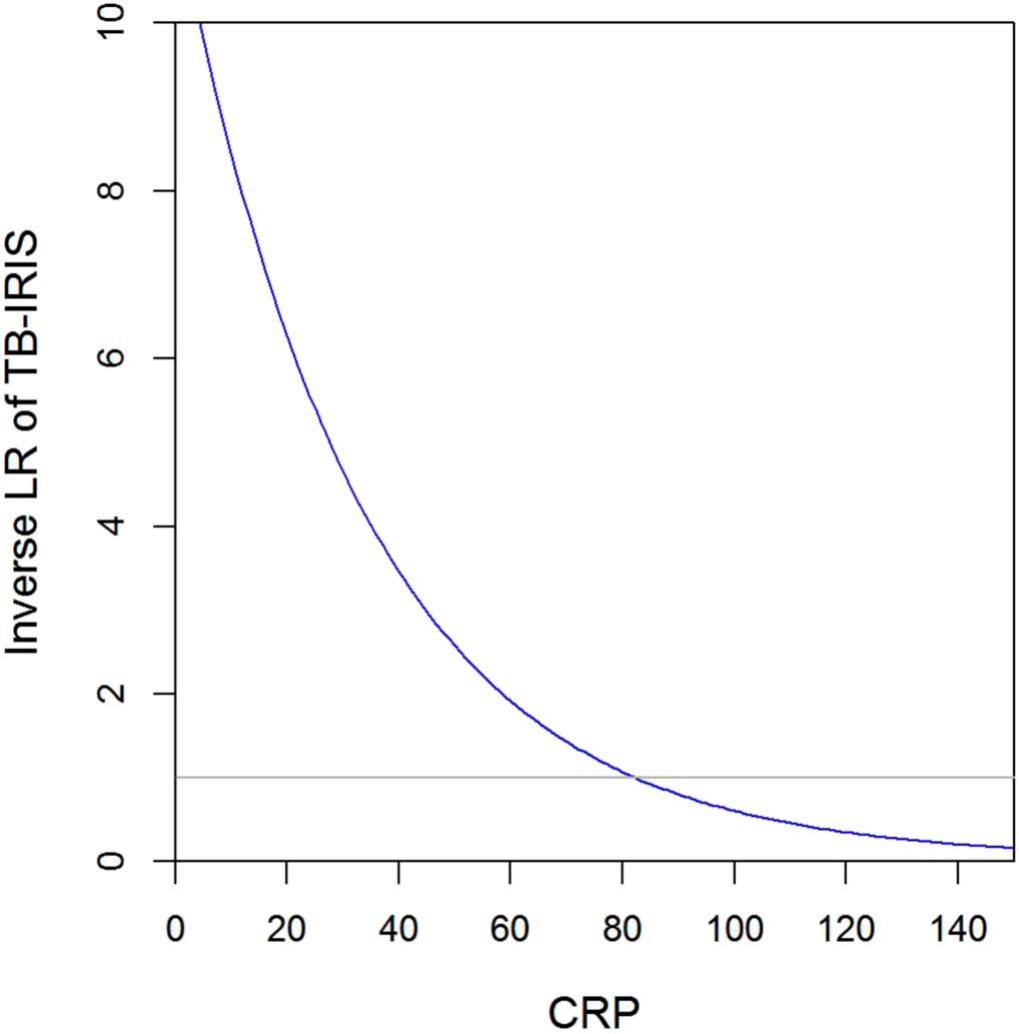
The association between CRP and likelihood of TB-IRIS. CRP (mg/L) is plotted against the inverse likelihood ratio of TB-IRIS, as predicted by the latent class model. The inverse likelihood ratio indicates how many times less likely each CRP value is associated with TB-IRIS.

Of the 240 participants in the PredART trial, complete data for latent class modeling were available for 217 participants (see Figure 1, Supplemental Digital Content, http://links.lww.com/QAI/B581). Baseline variables for these 217 were comparable with those of all participants in the PredART trial (data not shown).

### Presentation of Paradoxical TB-IRIS

Eighty-nine participants (41%) developed TB-IRIS according to the INSHI criteria. Twelve (6%) participants were adjudicated by the committee to have paradoxical TB-IRIS not fulfilling the INSHI criteria. Of the INSHI TB-IRIS cases, 66 (74%) fulfilled at least one INSHI major criterion, criteria 1 and 2 (new or enlarging lymph nodes or new or worsening chest CXR abnormalities) being the most frequent. None of the participants fulfilled INSHI major criterion 3 (neurologic features). All but one of the participants who developed TB-IRIS also fulfilled at least one minor criterion. Twenty-three participants (26%) had a diagnosis of TB-IRIS based on having 2 or more minor criteria without a major criterion. Of the participants without TB-IRIS, 26 (20%) fulfilled 1 minor criterion; this included all 12 participants assessed by the committee to have paradoxical TB-IRIS, but not fulfilling the INSHI criteria.

The most frequent symptoms in participants with INHSI TB-IRIS were respiratory symptoms (65%), night sweats (56%), loss of appetite (53%), and loss of weight (51%) followed by vomiting (38%), diarrhea or loose stool (31%), and weakness (34%). Fever (temp > 37.7°C) was present in 37% of the participants with INSHI TB-IRIS and tachycardia (heart rate > 120/min) in 70%. Sixty-two percent had a CRP > 90 mg/L (Table 1).

### Latent Class Model

The final latent class model included 9 variables: respiratory symptoms, night sweats, INSHI major criteria 1, 2, and 4 (new or enlarging lymph nodes, radiological abnormalities, and serositis, respectively), maximum CRP >90 mg/L, maximum heart rate >120/min, maximum temperature >37.7°C, and pre-ART CD4 count <50 cells/µL. The model showed a good fit to the data (χ^2^ = 337, *P* = 1.0). The model-estimated incidence of TB-IRIS was 43%. The predicted sensitivities and specificities of the variables included in the model are summarized in Table 2. Other models, for example, a model with the 5-model selected variables, a model including baseline urine LAM instead of maximum CRP, or a model including weight loss instead of fever showed similar results to the selected model (see Table 1, Supplemental Digital Content, http://links.lww.com/QAI/B581).

**TABLE 2.** LCA-Predicted Sensitivity and Specificity of Variables Included in the Final Latent Class Model

Variable	LCA-Predicted Sensitivity	LCA-Predicted Specificity
Respiratory symptoms	0.57	0.79
Night sweats	0.58	0.91
INSHI major 1 (new or enlarging lymph nodes)	0.28	0.96
INSHI major 2 (new or worsening CXR abnormalities)	0.38	0.96
INSHI major 4 (new or worsening serositis)	0.02	0.99
Maximum CRP > 90 mg/L	0.73	0.88
Maximum heart rate >120 bpm	0.79	0.78
Maximum temperature >37.7°C	0.44	1.00
Nadir CD4 count <50 cells/µL	0.81	0.54

### Comparison of Case Definitions

Using the model-predicted probability of TB-IRIS for each participant, we found the INSHI consensus case definition had a sensitivity of 0.77 and a specificity of 0.86. We constructed several adapted case definitions, replacing one or 2 of the required INSHI minor criteria with one or more of the model-derived variables CRP (>90 mg/L), heart rate (>120/min), or fever (temperature > 37.7°C). The adapted case definitions had sensitivities and specificities similar to the INSHI consensus case definition. A definition replacing all the minor criteria with objective measures (CRP elevation, fever, and/or tachycardia) showed better diagnostic accuracy, with a sensitivity of 0.89 and a specificity of 0.88. Performance of the INSHI case definition and the adapted case definitions to identify TB-IRIS is summarized in Table 3.

**TABLE 3.** Diagnostic Accuracy of INSHI and Adapted Case Definitions

TB-IRIS Definition	Sensitivity	Specificity	LR+	LR−
INSHI case definition: Presence of at least	0.77	0.86	5.50	0.27
1 INSHI major criterion OR				
2 INSHI minor criteria				
CRP >90 mg/L	0.73	0.88	6.08	0.31
Baseline urine LAM ≥1	0.76	0.67	2.30	0.36
Presence of at least 1 INSHI major criterion alone	0.60	0.92	7.50	0.43
Presence of at least	0.85	0.78	3.86	0.19
1 INSHI major criterion *OR*				
2 INSHI minors criteria *OR*				
1 INSHI minor criterion and CRP >90 mg/L				
Presence of at least	0.86	0.83	5.06	0.17
1 INSHI major criterion *OR*				
1 INSHI minor criterion and CRP >90 mg/L				
Presence of at least	0.93	0.82	5.17	0.09
1 INSHI major criterion *OR*				
1 INSHI minor criterion and one of the following:				
CRP >90 mg/L				
Heart rate >120/min				
Temperature >37.7°C				
Presence of at least	0.89	0.88	7.42	0.13
1 INSHI major criterion *OR*				
Two of the following:				
CRP >90 mg/L				
Heart rate >120/min				
Temperature >37.7°C				

Case definitions were constructed, and their sensitivity, specificity, and positive and negative likelihood ratios computed using the LCA-predicted probability of TB-IRIS for each participant.

LR+, positive likelihood ratio; LR−, negative likelihood ratio.

### CRP to Rule out TB-IRIS

Using the model-predicted probability of TB-IRIS for each participant, we found CRP values of > 10 mg/mL to > 50 mg/mL all had a sensitivity above 0.9 and a negative likelihood ratio of ≤ 0.15 (indicating a moderate to large decrease in probability of having a CRP value lower than the cut-off when having TB-IRIS) (see Table 2, Supplemental Digital Content, http://links.lww.com/QAI/B581). The area under the ROC curve was 0.86. The association between CRP and likelihood of TB-IRIS is shown in Figure 1, showing the inverse likelihood ratio, which indicates how many times less likely each CRP value is associated with TB-IRIS. We repeated the analysis using an alternative model including baseline urine LAM instead of CRP to confirm our findings; this analysis showed similar results (data not shown).

## DISCUSSION

Because of the lack of a gold standard, validation of the INSHI consensus case definition for paradoxical TB-IRIS has previously been performed against diagnostic assignment based on expert opinion^4,5^ or another expert opinion–based case definition,^6^ showing sensitivities of 0.72–0.91 and specificities of 0.93–1.0. We applied LCA, using participant data from a prospective trial of a TB-IRIS prevention strategy, to provide a surrogate gold standard for TB-IRIS. Participants included in this trial were all coinfected with HIV and TB and starting ART. This surrogate gold standard enabled us to validate the INSHI consensus case definition and confirm its reasonable diagnostic accuracy found in the previous studies using a data-derived approach.

The INSHI consensus case definition consists of 3 components: a prerequisite of a diagnosis of TB with an initial positive response to treatment, clinical features summarized in a combination of major and minor criteria, and exclusion of other reasons for clinical deterioration. The first and the last component are largely unambiguous. However, many of the INSHI minor criteria have a subjective component and depend on patient-reported symptoms. Replacing these with objective variables could make the definition more robust and improves uniformity, which is advantageous if the definition is to be used for endpoint definition in clinical trials. We found that amending the case definition by replacing the INSHI minor criteria with the more objective variables tachycardia, fever, and/or CRP elevation improved sensitivity without loss of specificity.

CRP in itself may have utility as a rule-out test for TB-IRIS. We assessed this diagnostic ability of CRP and found that a normal CRP (<10 mg/L) can be used to rule out TB-IRIS. Because most patients with HIV-associated TB will however have a CRP > 10 mg/L,^14–16^ we also assessed higher CRP cut-offs. Figure 1 showed that especially a low CRP is useful in excluding TB-IRIS, but higher cut-off values of CRP still have an added value in ruling-out TB-IRIS. A CRP value of 82.4 corresponds to the cut-off that is equally likely for patients with or without TB-IRIS.

Our study has several limitations: first, a relatively small study cohort and inclusion of a large number of possible variables in the latent class model resulted in the need to preselect variables based on their univariate association with TB-IRIS so as not to exceed 10 variables in the model. Consequently, some important variables may have been excluded. However, we explored different selection criteria which resulted in similar results giving assurance of the robustness of the model. Second, there were no participants with a temperature above 37.7°C among those who were classified as not having TB-IRIS according to the latent class models. Leaving temperature out of the model did not affect this finding. Looking at the raw data, however, only 4 of the 41 participants with a temperature above 37.7°C did not have TB-IRIS, according to either the INSHI case definition or the adjudication committee; all 4 had a more likely alternate diagnosis (gastrointestinal infection, drug resistant TB, drug rash, and drug-induced liver injury). Third, in the PredART trial, CXRs were only repeated on ART when suspected TB-IRIS or other clinical deterioration prompted the clinician to request a CXR. As a consequence, we do not have documented normal CXRs for all participants who did not develop TB-IRIS, and therefore cannot say with certainty that none of the participants in this group had new or worsening CXR features. However, in the absence of other symptoms, one can question its clinical relevance. Fourth, this was a restricted patient population, purposefully selected for its high risk of TB-IRIS; only patients with a CD4 count ≤100 cells/µL and antituberculosis treatment for <30 days before starting ART were included in the trial. It could be our model performs differently in a population with a lower incidence of TB-IRIS. Moreover, our findings only apply to patients with HIV-associated TB starting ART and not to paradoxical reactions that may occur in HIV-negative patients undergoing immune reconstitution for other reasons. Fifth, the method we used allows for only the estimation of the diagnostic indicators of variables included in the model and other variables. Calculation of the uncertainty and resulting confidence intervals of these estimates is however complex and is hampered by the inherent multiplicity because of variable selection in LCA model building. For this reason, we did not estimate confidence intervals for diagnostic accuracy measures in this analysis. Consequently, these estimates should be seen as exploratory and will need validation in an independent data set to correctly estimate the bias and uncertainty in these estimates.

In conclusion, we found that the INSHI case definition identifies TB-IRIS with reasonable accuracy. Amending the case definition by replacing the INSHI minor criteria with the objective variables tachycardia, fever, and/or CRP elevation improved sensitivity without loss of specificity in a population at a high risk of TB-IRIS. We recommend that in future studies on TB-IRIS a version of the INSHI case definition with objective measures be used, next to the traditional case definition. CRP seems to be promising as a test for ruling out TB-IRIS.
